# A novel tumor immunotherapy-related signature for risk stratification, prognosis prediction, and immune status in hepatocellular carcinoma

**DOI:** 10.1038/s41598-023-46252-3

**Published:** 2023-10-31

**Authors:** Jianping Sun, Lefeng Xi, Dechen Zhang, Feipei Gao, Liqin Wang, Guangying Yang

**Affiliations:** Department of Pathology, Zhengzhou YIHE Hospital, Zhengzhou, 450000 Henan Province China

**Keywords:** Cancer immunotherapy, Cancer microenvironment, Gastrointestinal cancer

## Abstract

Immunotherapy as a strategy to deal with cancer is increasingly being used clinically, especially in hepatocellular carcinoma (HCC). We aim to create an immunotherapy-related signature that can play a role in predicting HCC patients’ survival and therapeutic outcomes. Immunotherapy-related genes were discovered first. Clinical information and gene expression data were extracted from GSE140901. By a series of bioinformatics methods to analyze, overlapping genes were used to build an immunotherapy-related signature that could contribute to predict both the prognosis of people with hepatocellular carcinoma and responder to immune checkpoint blockade therapy of them in TCGA database. Differences of the two groups in immune cell subpopulations were then compared. Furthermore, A nomogram was constructed, based on the immunotherapy-related signature and clinicopathological features, and proved to be highly predictive. Finally, immunohistochemistry assays were performed in HCC tissue and normal tissue adjacent tumors to verify the differences of the four genes expression. As a result of this study, a prognostic protein profile associated with immunotherapy had been created, which could be applied to predict patients' response to immunotherapy and may provide a new perspective as clinicians focus on non-apoptotic treatment for patients with HCC.

## Introduction

Among the various solid tumors in humans, liver cancer is one of the tumors that has attracted much attention, and one of the world’s deadliest cancers. This disease is often at an advanced stage when diagnosed, which is the main cause for its high mortality^1^. Thus, further research is needed to identify key molecules in the progression of hepatocellular carcinoma and guide clinicians in making accurate diagnosis and treatment decisions. Immunotherapy was used in many disease areas with impressive results and revolutionized cancer treatment^2^. Such as lung cancer^3^, pancreatic cancer^4^, medulloblastoma^5^, and etc. it had emerged as an invaluable treatment option for many patients with HCC^6,7^. However, they were not successful in all patients. Despite these advances in the understanding of treatment strategy, the key molecules that influence the efficacy of HCC immunotherapy need to be further clarified.

Proteins are the basic functional units of the human body, and different proteins have their unique roles, for example, as enzymes, antibodies, messengers^8,9^, etc. For the treatment of tumors, they are often present as targets, such as PD-L1 is an important immune checkpoint^10^. In the present study, a new prognosis-related risk model about immunotherapy-related proteins was developed, it can be a potential target for individual immunotherapy as well as a prognostic indicator for HCC.

## Materials and methods

### Collect public data and identify immunotherapy-related genes

We downloaded the microarray data of HCC patients after PD-1/PD-L1 immunotherapy from the GSE140901 database in NCBI (https://www.ncbi.nlm.nih.gov/geo/)^11^. Other RNA sequencing data (RNA-seq) and clinicopathology features data used in the paper were gained from three free and publicly available datasets: TCGA-LIHC (https://xena.ucsc.edu), ICGC (LIRI-JP) (https://dcc.icgc.org). We obtained some images in public databases, protein expression in HPA (http://www.proteinatlas.org) and cell line expression in CCLE (http://www.ccle.org)^12^. Table S1 shows the general clinical characteristics of HCC patients from the database and the author’s hospital.

### WGCNA and co-expression modules analysis

After all genes were included in the Weighted correlation network analysis (WGCNA) process, power values were filtered out as each module was built. In the gradient method, each module had a different power value (ranging from 1 to 20) was tested for independence and average connectivity. It was subsequently found that the power value reached most appropriate when the degree of independence was close to 0.85. To make the results highly reliable, we extracted the gene information corresponding to each module, and 50 was the minimum number of genes we set. "Heatmap 3" further determined the interaction of co-expression modules.

### Creation of an immunotherapy-related signature

Univariate Cox regression with *p* < 0.05 was our method used to filter out genes related to patient’s prognosis after immunotherapy. The screened prognostic genes were then screened for the most valuable genes using least absolute shrinkage and selection operator (LASSO) Cox regression, random forest algorithm and stepwise COX regression models, and overlapping genes were further incorporated into a multivariate Cox regression model to establish an immunotherapy-associated signature. The multivariate Cox relapse coefficient (β) was applied to build a risk score found on directly mixing the equation below with genes expression levels. Risk score = ∑iCoefficient (genes)*Expression (genes). In the GSE140901 dataset, the ROC (receiver operating characteristic curve) curve was used to assess the diagnostic accuracy of this signature. Furthermore, in the Metascape database (https://metascape.org), gene set enrichment analysis (GSEA) was performed to separate the altogether cautious GO and KEGG items based on different scores^13^. At last, a nomogram model was established to study the predictive accuracy of the signature in TCGA database.

### Immune infiltrate and genetic alterations analysis

To obtain the abundance ratio of infiltrating immune cells in the tumor immune microenvironment, we used TIMER^14^, CIBERSORT^15^ and xCELL^16^ databases. Mutation data were acquired from TCGA, and genetic variation in different subgroups was assessed using the R package "maftools".

### IHC assays for protein levels of genes within the immunotherapy-associated signature

Four antibodies were bought back from Thermo Fisher Scientific. Immunohistochemistry was used to detect 40 HCC tumor tissues and 40 adjacent normal tissues from YIHE Hospital. After informing all patients of the purpose of our study, all patients signed a written informed consent form for the organization’s donation. These tissues were first fixed with formalin, then embedded with paraffin, and finally made into 3 μm thick sections by a sectioning mechanism for later use. After antigen repair, the sections were incubated overnight at 4 °C with either ENG (PA5-79203; 1:200 dilution), FCER1G (PA5-28832; 1:500 dilution), PSEN1 (PA5-98093; 1:100 dilution), SLAMF6 (MA5-29572; 1:500 dilution), and binding was detected using the avidin–biotin–peroxidase method. Block slides after hematoxylin counterstaining. It was subsequently referred to two experienced pathologists for an independent, double-blind evaluation. The immunohistochemical positive intensity score criteria were graded as 0, 1, 2, and 3 for no, weak, moderate, and strong staining, respectively. Scores of 0 and 1 were considered low expressions, while scores of 2 and 3 were high expressions.

### Statistical analysis

An independent-sample t-test was used to analyze quantitative variables. With R software (version 4.0.3), ROC curve analysis and Kaplan–Meier survival analysis were used to evaluate the accuracy of predicting survival outcomes. Cox proportional models were used to examine the relationship between prognostic classifiers and survival outcomes, as well as other clinical parameters. The results were considered statistically significant when the P-value was less than 0.05.

### Ethics approval and consent to participate

This study was supported by the Ethics Committees of YIHE University. Written informed consent was obtained from all patients. All methods were performed following the relevant guidelines and regulations. The Ethics Number: YH-LL-KY00101.

## Results

### WGCNA and identification of immunotherapy-related genes

Using cluster analysis, we checked the quality of the data from 24 samples, and one sample in the cohort was removed from our survey (Fig. S1A). Then, a network was constructed based on clinical data from 23 samples of HCC using the WGCNA. After setting the soft threshold power β to 9, we constructed a scale-free network, and the independence degree was 0.85. (Supplementary Fig. S1B).

**Figure 1:**
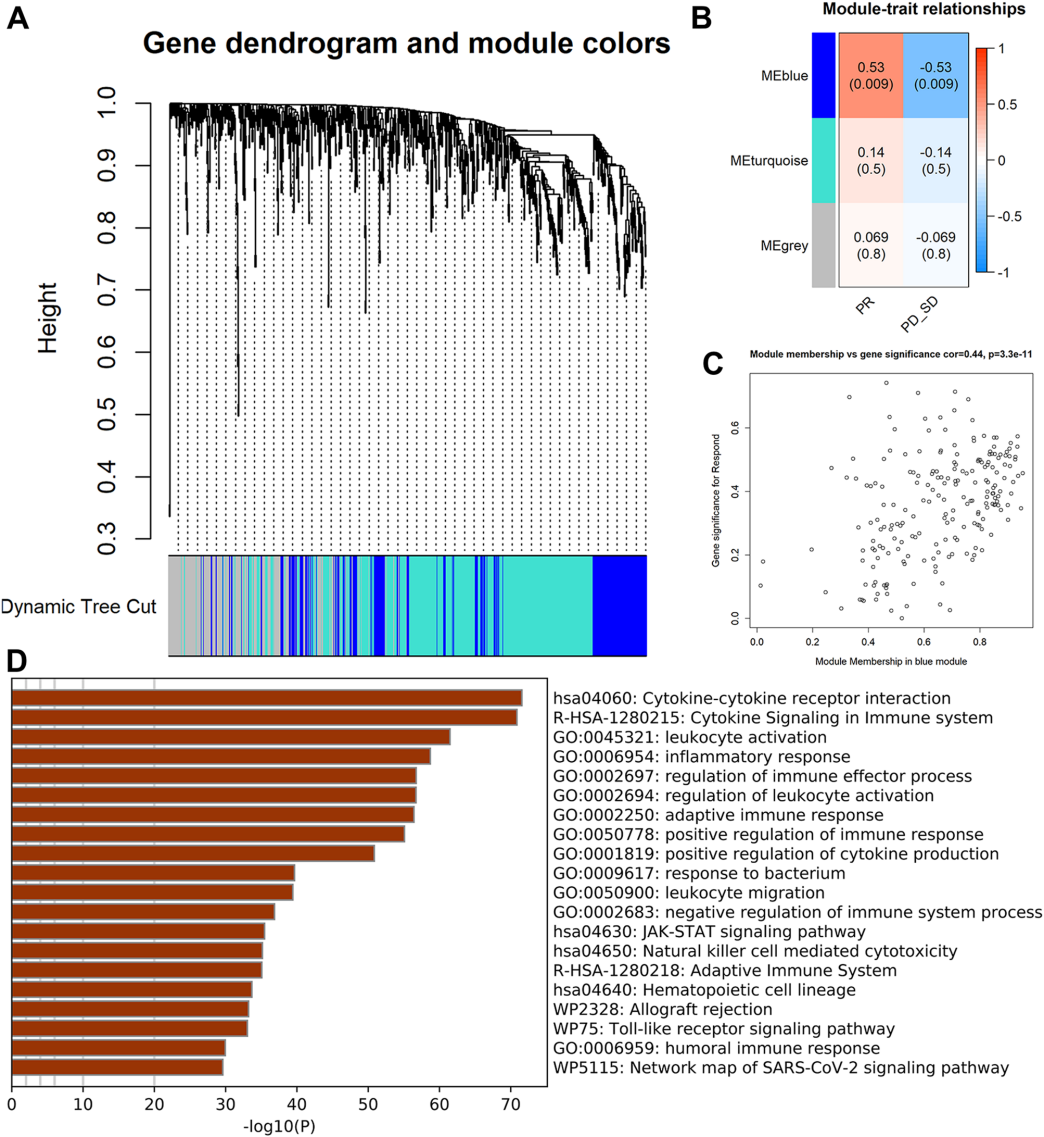
Identification of genes related to immunotherapy treatment. (**A**) As a result, two non-grey modules are filtered out. (**B**) The blue module was significantly associated with immunotherapy efficacy. (**C**) The scatter plot for genes in the blue module of GS score and MM. (**D**) Enrichment analysis of Immunotherapy-related genes from the blue module.

Modules of immunotherapy-related genes with similar expression patterns clustered together, and modules with a cut height difference of < 0.25 were merged. This process results in three co-expression modules: blue, turquoise, and grey (Fig. 1A, B). The blue module was the only one to strongly correlate positively with immunotherapy efficacy. MM and GS scores were strongly correlated in the blue module (Fig. 1C). Therefore, in the blue module, 207 genes were analyzed for hub genes. KEGG analysis showed that genes were principally enriched in the following signaling pathways: cytokine signaling in the immune system, cytokine receptor interaction, Natural killer cell-mediated cytotoxicity and adaptive immune system. GO analysis showed that genes were principally enriched in the following biological processes: immune cell activation, humoral immune response, and immune response regulation (Fig. 1D).

### Create and verify immunotherapy-related signatures

**Figure 2:**
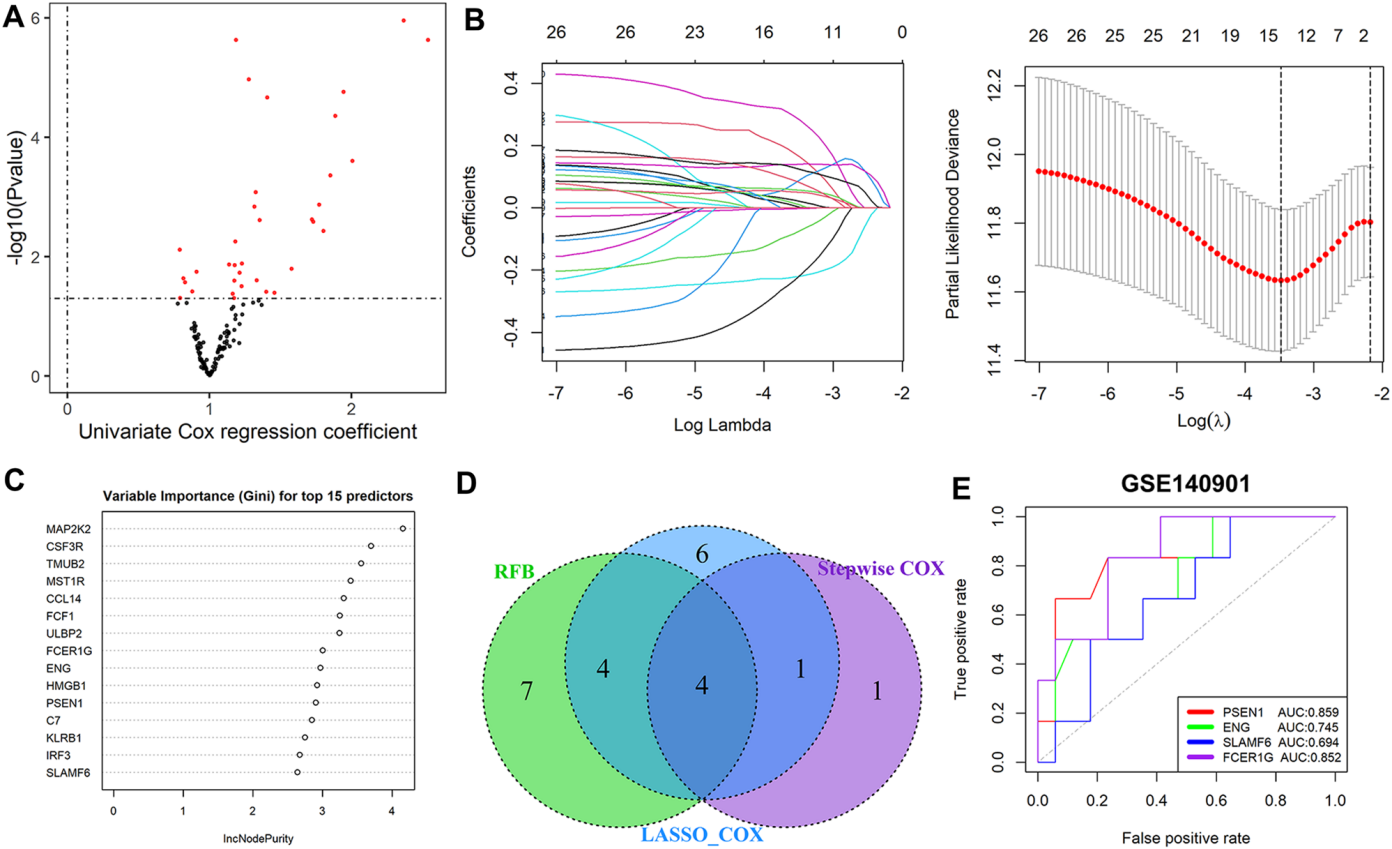
Identification of genes related to immunotherapy treatment. (**A**) Immunotherapy-related genes by univariate Cox regression. (**B**) Immunotherapy-related genes screened by the LASSO-Cox regression model. (**C**) Immunotherapy-related genes screened by the Random forests model. (**D**) Four overlapping genes were considered Immunotherapy-related genes. (**E**) Validation of the diagnostic value of immunotherapy Efficacy in the dataset GSE140901.

**Figure 3:**
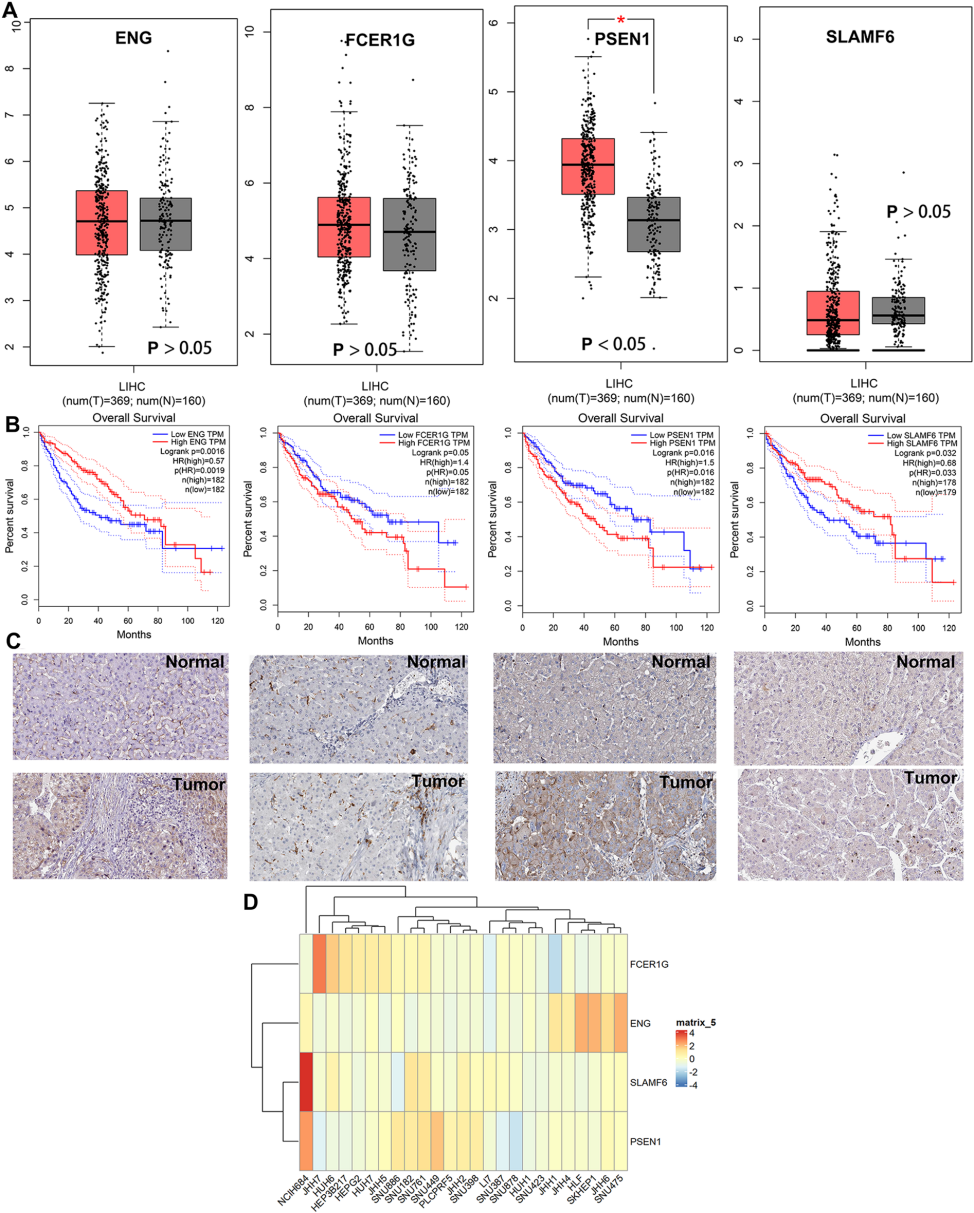
Expression and prognostic significance of the 4 genes. (**A**) Expression difference in the tumor and normal tissues. (**B**) Prognostic value analysis. (**C**) Protein’s expression levels in the tumor and normal tissues. (**D**) Gene’s expression levels in the cell lions.

A total of 28 candidate genes with a *P* < 0.05 were determined to be related to patient prognosis (Fig. 2A), further screening by LASSO-Cox regression model (Fig. 2B), Random forests model (Fig. 2C), and Stepwise COX regression model. Then, the overlapped genes were gained by the Veen model, and a four-genes signature was finally created (Fig. 2D). To assess the potential value for diagnosing of the four-genes, the ROC curve had been used, and had a good predictive power, and the AUCs were 0.859, 0.745, 0.694, and 0.852 in the datasets GSE140901, respectively (Fig. 2E). The results from database showed that only the PSEN1’s expression level was different in tumor and normal tissues (Fig. 3A), although all the four genes were closely associated with patient’s prognosis (Fig. 3B), according to the results of GEPIA database (http://gepia.cancer-pku.cn)^17^. The expression profiles of ENG, FCER1G, PSEN1, SLAMF6 from HPA (http://www.proteinatlas.org) shown that, ENG, FCER1G, SLAMF6 were not expressed or lowly expressed in tumor and normal tissues; PSEN1 was unexpressed or underexpressed in normal tissues and highly expressed in tumor tissues (Fig. 3C). We obtained the expression levels of 4 genes in HCC cell lines from the CCLE database (http://www.ccle.org), shown in Fig. 3D.

### Establishment of an immunotherapy-related genes signature in TCGA

**Figure 4:**
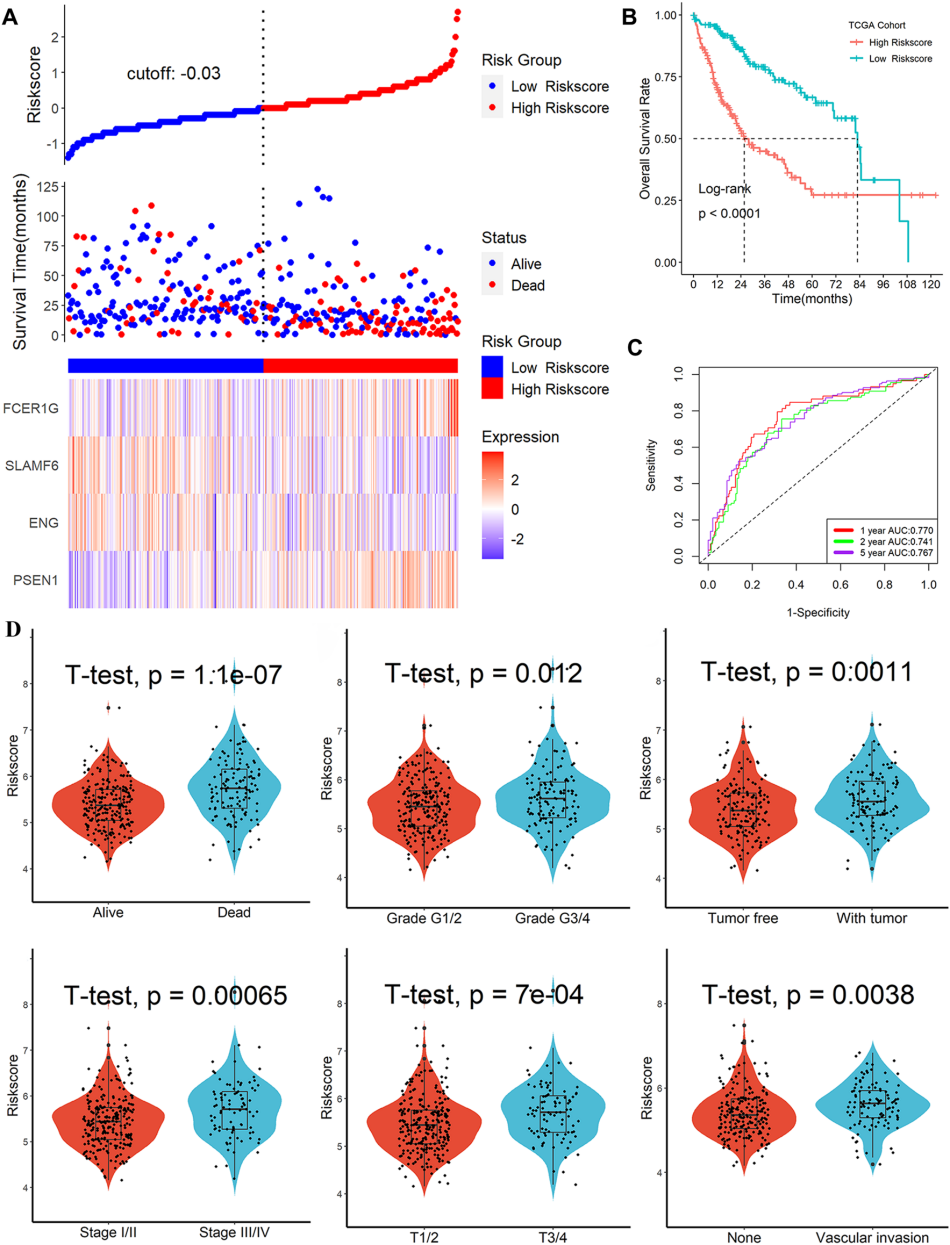
Construction of Immunotherapy-related genes signature in TCGA. (**A**) Risk score distribution, OS status and expression profile of four genes. (**B**) In the TCGA cohort, survival outcomes were significantly decreased in patients with higher risk scores. (**C**) ROC analysis predicts the prognostic value of 1-, 2-, and 3-year OS rates. (**D**) Higher risk scores were linked to different survival status, such as grade, recurrence status, TNM stage, T stage, and vascular invasion.

These four genes were placed into multivariate Cox-regression model, then, a 4-gene signature related to HCC prognostic was identified. Risk score = PSEN1*0.7484834-ENG*0.2827324-SLAMF6*0.4829894 + FCER1G*0.6202788. After calculating the risk score of TCGA liver cancer patients using the above formula, patients were divided into two risk subgroups based on the optimal risk score threshold (Fig. 4A). Kaplan–Meier survival analysis showed that the prognosis was better when the score was lower, while those with higher scores had the opposite outcomes (Fig. 4B), and ROC analysis found that this signature got an ideal predictive function for patient prognosis with AUCs at 1-, 2-, 3-year of 0.770, 0.741, 0.767(Fig. 4C). As shown as Fig. S2, differential gene enrichment in the high- and low-risk subgroups in immune-related biological processes. Our following job reveals that living patients had lower risk scores than dead patients. Even more, patients in the advanced clinical stage (Fig. 4D) always had a higher risk score. This information suggests that patients with low-risk score have a better outcome.

### Verification of the signature in the ICGC

For validating the signature, ICGC datasets were token into use as validation cohort. The same formula was used to calculate a patient's risk score. In the ICGC, patients were divided into high-risk and low-risk subgroups (Fig. S3A) in the ICGC dataset, we found that surviving patients with hepatocellular carcinoma (Fig. S3B) or in the early stage of TNM were accompanied by lower scores (Fig. S3C). The results of the Kaplan–Meier survival analysis showed that in the ICGC cohort, the higher the risk score, the lower the OS rate, and the two were significantly negatively correlated (Fig. S3D). ROC analysis showed that the prognosis prediction of this signature was also very good in the ICGC cohort, with AUC of 0.659, 0.634, and 0.656 at 1, 2, and 3 years, respectively (Fig. S3E).

### Immune infiltrate and genetic alterations analysis

**Figure 5:**
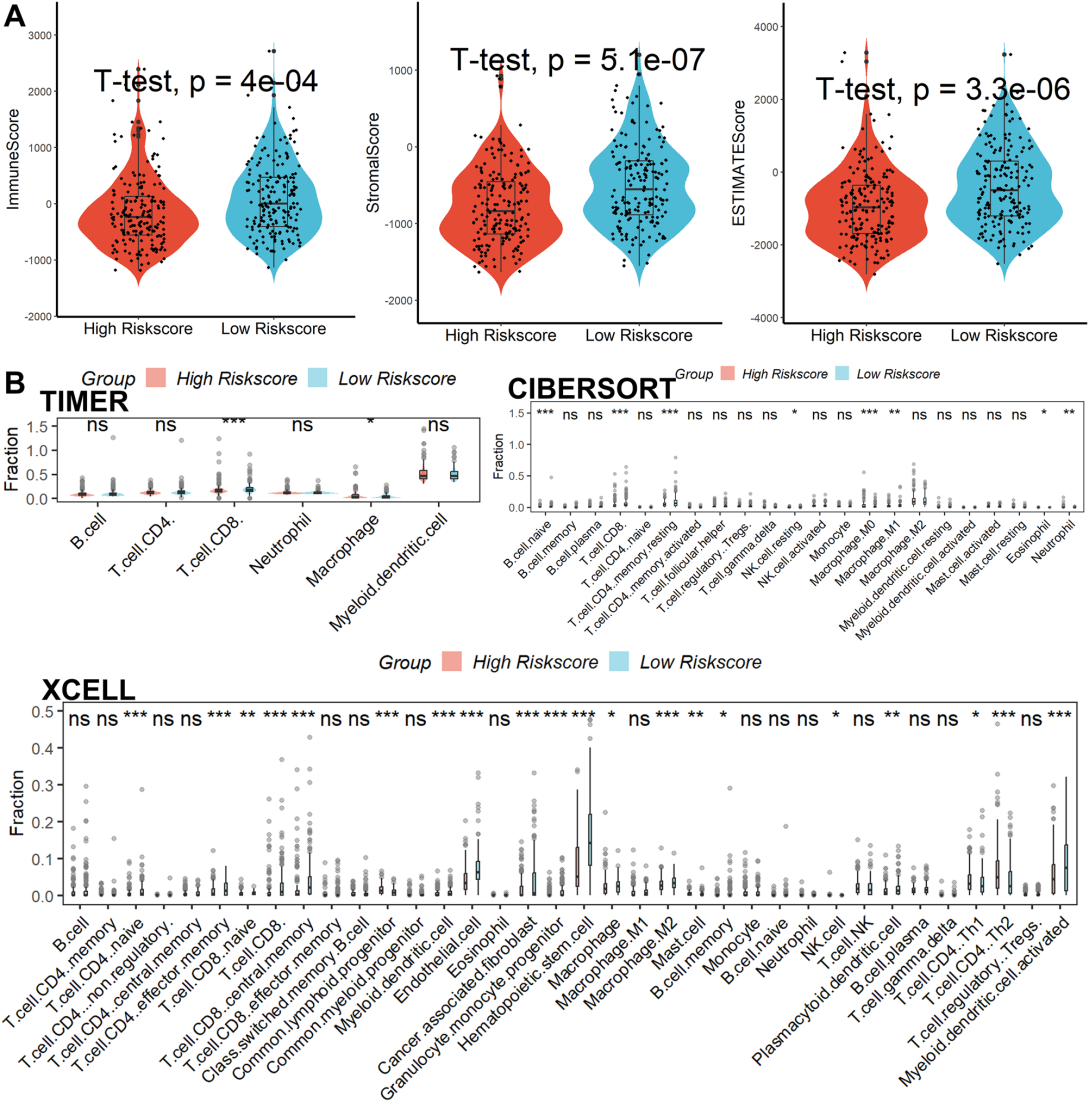
Immune infiltrate analysis. (**A**) Risk score was significantly correlated with immune score, interstitial score, and estimation score. (**B**) A heatmap of all substantially different immune cells between the high- and low- score subgroups.

**Figure 6:**
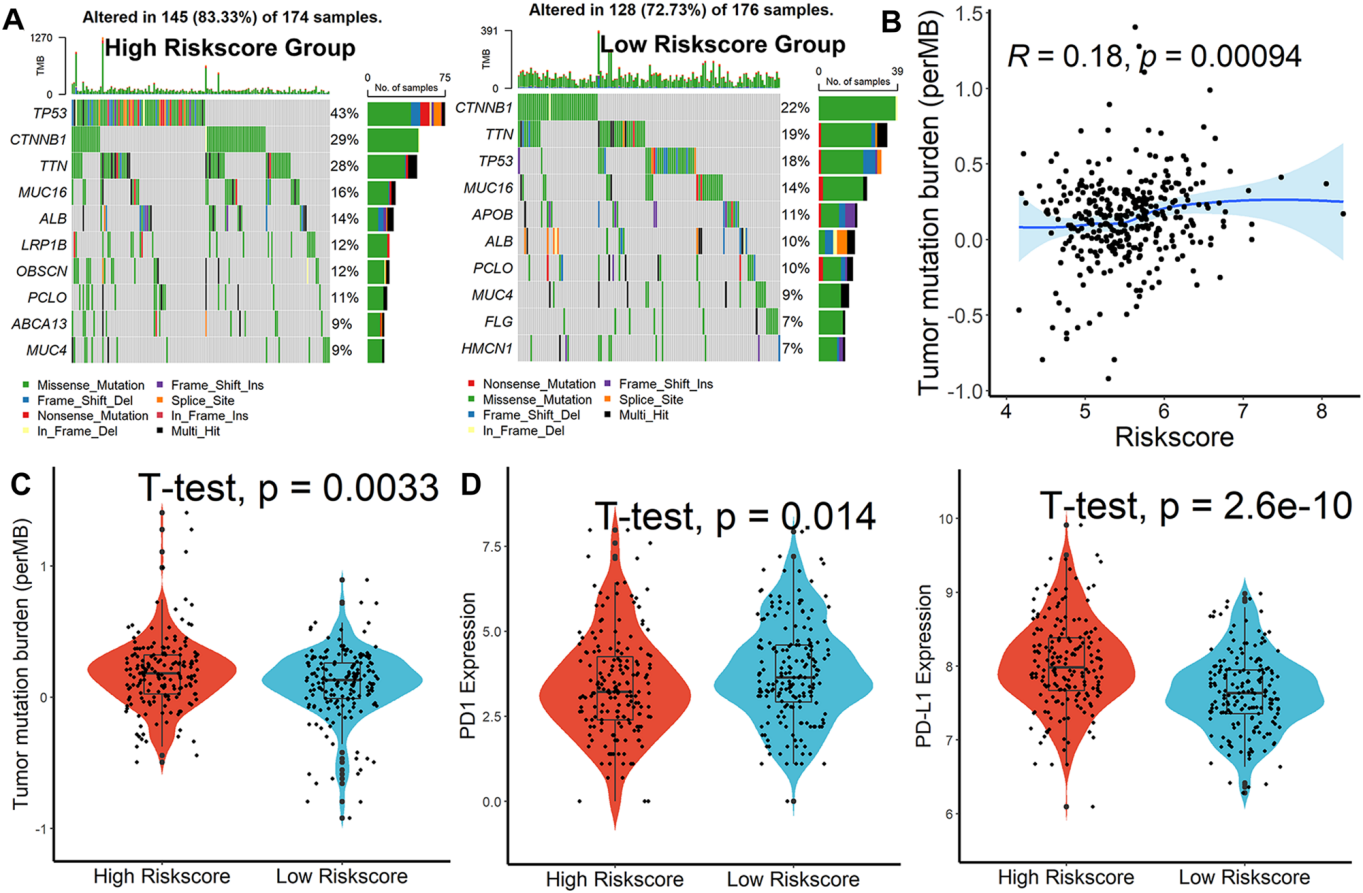
Genetic alterations, TMB, and immune infiltrate analysis. (**A**) Top 10 genes with the highest mutation rates in the high-, and low-risk score subgroups. (**B**) Correlation, and differential (**C**) analysis of risk scores and TMB. (**D**) Analysis of differences between risk scores and PD1, PDL-1 expression.

**Figure 7:**
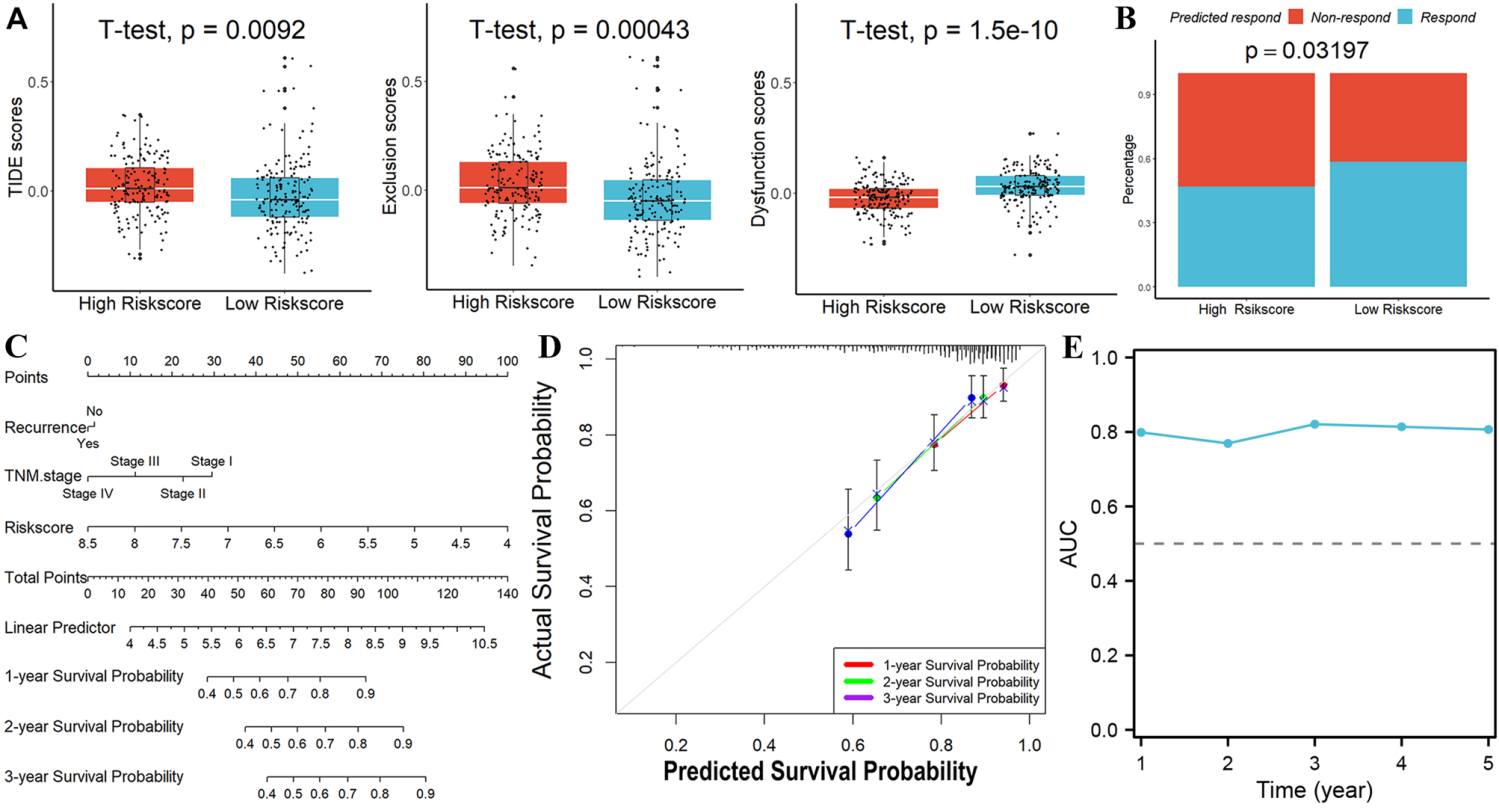
Validation of the predictive significance of immunotherapy-related gene signatures in nomogram model. (**A**) Nomogram combining the 4 genes signatures. (**B**) Calibration plots of 1-, 2-, and 3-year survival probabilities.

Significant difference in ImmuneScore was observed between the two groups. The high-score group had lower scores, Stromalscore and ESTIMATEscore observed same results (Fig. 5A). After using TIMER, CIBERSORT, and xCELL databases to determine the abundance ratio of infiltrating immune cells in the immune microenvironment of HCC tissues, a heat map of all the different immune cells was made. In the TIMER database, we found that the infiltration level of CD8^+^ T cells was remarkably reduced in the high-risk group. The results we obtained from CIBERSORT revealed that the infiltration levels of CD8^+^ T cells, CD4^+^ T cells, Macrophage.M1 cells, Macrophage.M2 cells were significantly lower in the high-risk score group. The results we obtained from xCELL showed that the infiltration levels of Myeloid dendritic cell activated cells, CD8 + naïve T cells, Common lymphoid progenitor cells, CD8 + central memory T cells, Endothelial cells, Hematopoietic stem cells, Macrophage cells, Macrophage.M2 cells, Plasmacytoid dendritic cells, CD4^+^ Th1 T cells, CD4^+^ Th2 T cells were significantly lower in the high-risk score group (Fig. 5B). All these results revealed that a decrease infiltration levels of CD8^+^ T cells in tumors. Analysis of genetic variation showed that the mutation rates of the top 10 genes with the most significant mutations differed significantly between two subgroups (Fig. 6A). Subsequently, the results of the TMB assessment for each patient showed a positive correlation between risk score and TMB (Fig. 6B, C). Next, higher levels of PD-L1 expression were detected in tumor tissue from high-risk group patients, suggesting that patients would benefit more from immune checkpoint inhibitors (ICIs) treatment in this group, even with lower PD1 expression levels (Fig. 6D). These suggest that the higher the risk score in our model, the worse the response to immunotherapy is likely to be. Furthermore, we used the TIDE scoring system to demonstrate this. As shown in Fig. 7A–B, in our model, the higher-risk group has a higher TIDE score, tumor cells are more prone to immune escape, and the outcome of the response to immunotherapy may be worse.

### Establish a nomogram model in TCGA

In TCGA, a nomogram model was developed to examine the coefficient prediction efficiency of this signature. Results indicated that the nomogram(C-index = 0.739) provided an accurate quantitative prediction method for predicting 1-to-3-year survival rate (Fig. 7C). From the overlap of predicted probability and actual probability of 1-to-3-year survival rate in the calibration curve, it showed great agreement (Fig. 7D). The AUC (area under the curve) values from these curves show that our model has good predictive potential (Fig. 7E).

### IHC assay in clinical samples

**Figure 8:**
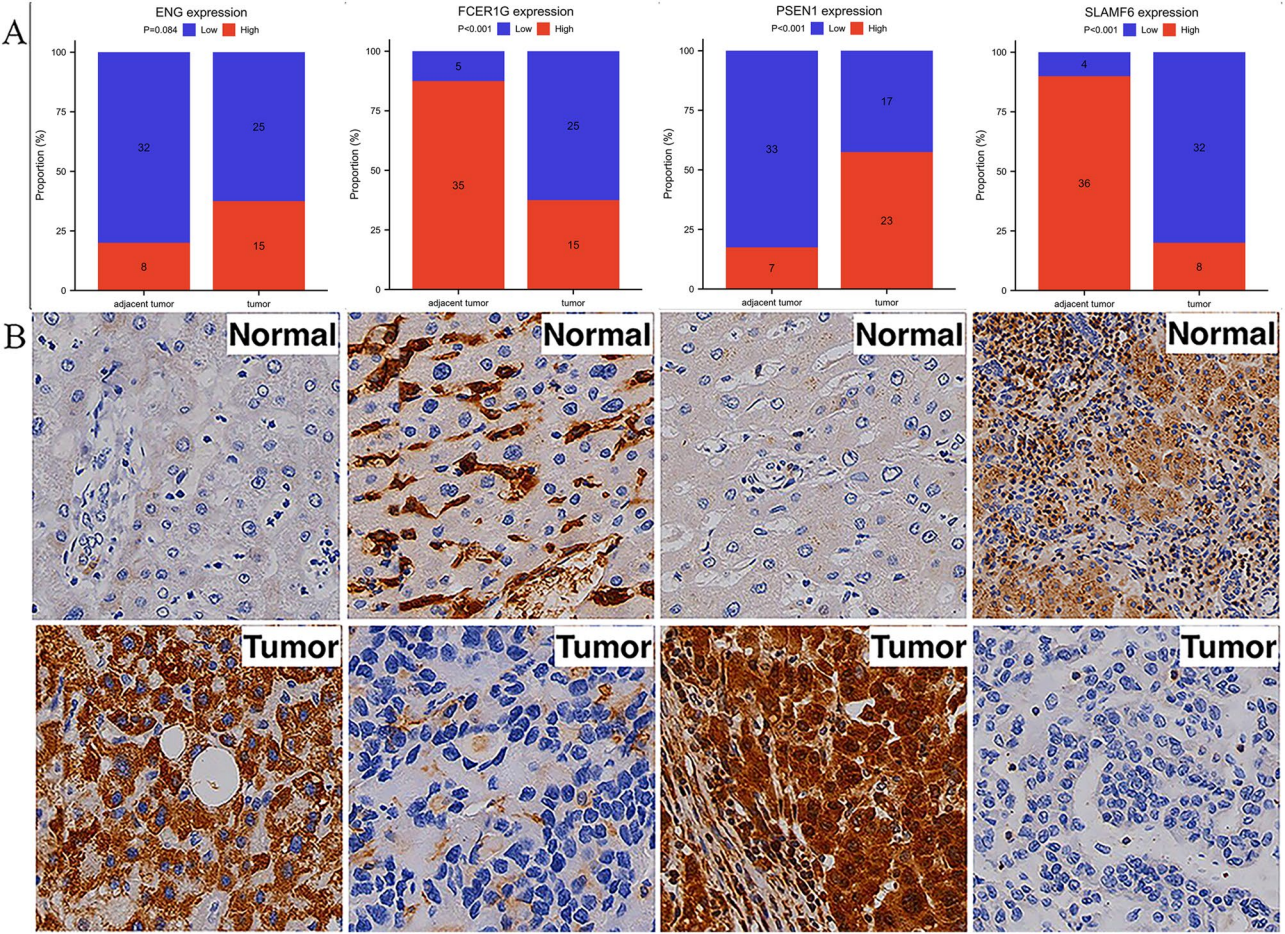
Protein’s expression in validation test cases. (**A**) Number of high and low expression cases in normal tissue adjacent tumor and tumor. (**B**) Protein’s expression levels in validation cases.

As demonstrated in Fig. 8, there was no difference in the expression level of ENG in tumor and normal tissue adjacent tumors, and the expression of FCER1G was lower in tumor tissue, PSEN1 was higher in tumor tissue, and SLAMF6 was lower in tumor tissue, with Fisher’s precision probability test results at (*p* = 0.084, *p* < 0.001, *p* < 0.001, and *p* < 0.001), respectively.

### Comparison with the previous signature

We tried to compare the predictive potential of several genetic signatures to help researchers better understand the prognostic significance. As shown in Fig. S4A–B, our signature showed better predictive power when using fewer genes than other previously published signatures.

## Discussion

Hepatocellular carcinoma was the second most common lethal tumor worldwide and had a high mortality rate. Compared with other types of liver cancer, HCC accounted for 90% of primary liver malignancies^1^. At present, in addition to liver transplantation, there are many options for treatment, mainly radical hepatectomy, local ablation, chemoembolization, transcatheter therapy, targeted therapy, etc.^18,19^ In the beginning, immune checkpoint inhibitor to be clinically studied in HCC was Tremelimumab, which targets CTLA-4^20^. Subsequently, it was confirmed in multiple research reports that immunotherapy was effective for liver cancer, and the immunosuppressive combination therapy developed immediately after that further improved the prognosis of patients^21–24^. Clinicians wishing to learn more about immunology were also working to tailor treatments to individual patients based on predictive biomarkers and etiology, potentially reshaping many previous treatment options and guidelines for the benefit of patients with advanced disease. Moreover, these strategies had been applied to the treatment of various cancers, such as melanoma, lung cancer, glioblastoma, etc. However, to date, few patients have received long-term remission, and only 20–40% of patients have responded to immunotherapy. How to select appropriate detection and prognostic biomarkers and screen the most beneficial patient population are both opportunities and challenges.

Now, we constructed a new 4-gene prognostic risk model found on immunotherapy, which had high accuracy in predicting prognosis of HCC patients in the TCGA and ICGC sets. What is more, study had shown that this signature significantly affected the immune microenvironment as well as response to immune checkpoint inhibitors in HCC. In the subsequent mutation gene analysis, we found that TP53, CTNNB1 and TTN gene mutations were predominant in patients in the high-risk group, while the mutation rates of these three genes were lower in the low-risk group. Mutations in TP53 and CTNNB1 genes are common oncogenic factors and are usually associated with poor tumor prognosis. A higher proportion of these genes’ mutation in the high-risk group also predicts a worse prognosis for tumor patients. In addition, LRP1B, OBSCN and ABCA13 high-frequency mutated genes were also present in the high-risk group, while APOB, FLG and HMCN1 high-frequency mutated genes were alone in the low-risk group, and the two groups had significantly different gene mutation patterns. patients with high-risk scores were accompanied by high TMB and higher levels of PDL-1 expression, suggesting an immune microenvironment of HCC could be affected by four-gene, and having shown that immunotherapy would be more effective in the high-risk score subgroup. By using TIMER, CIBERSORT and xCELL, we obtained results that CD8^+^ expression levels were lower in the high-risk group, which may be associated with T cell exhaustion in advanced tumors^25^. T cells were in constant contact with tumor antigens and become blunted in their reactivity. When tumor immune escape occurs, it often indicates that the body's defense ability to the tumor is reduced, resulting in a worse prognosis. Our analysis showed that the high-risk group had higher TIDE scores, higher T-cell Exclusion scores, and higher tumor immune dysfunction scores, which reduced the response rate to immunotherapy in this group and resulted in poorer outcomes. Finally, the nomogram model validated our risk model, which did have good predictive performance. Therefore, the use of the four-gene model we developed in clinical work related to immunotherapy can accurately predict the survival outcome and guide the selection of treatment.

According to the statistical results of the TCGA transcriptome, FCER1G and PSEN1 were negatively correlated with patient prognosis, while ENG and SLAMF6 were positively, and we performed further experimental verification. Endoglin (ENG) encodes homodimeric transmembrane proteins, as the main glycoprotein in the vascular endothelium, which is a component of transforming growth factor receptor complexes. This protein has a high affinity for BETA1 and BETA3 peptides and is involved in the regulation of angiogenesis. It is not only essential for the integrity and normal structure of the adult vasculature, but also regulates the migration of vascular endothelial cells. Work in recent years has shown that Endoglin may be a reliable disease biomarker and therapeutic target, which has brought it a lot of attention^26–28^. Our findings showed no significant difference in ENG expression levels in normal tissues from tumors and adjacent tumors. This result was consistent with those obtained in the TCGA database, but inconsistent with the results of some studies. Chen et al.^29^ showed that ENG is directly regulated by miR-370 and promote the occurrence and development of endometrioid carcinoma. HCC tumor tissue contains abundant microvessels, and tumor cells might express ENG protein to promote intertumoral angiogenesis, which will promote intrahepatic and extrahepatic metastasis of the tumor and presumably bring a worse prognosis, but further trials are needed. Function of the high-affinity IgE receptor (FCER1G) is associated with anaphylaxis. It is a tetramer consisting of 1 α, 1 β, and 2 γ chains. γ chain is also a subunit of other Fc receptors. It is a component of the high affinity immunoglobulin E (IgE) receptor involved in the transduction of allergic inflammatory signals in mast cells. It had been proved that it was expressed in monocytes/macrophages of tumor microenvironment^30,31^. Studies had shown that the subgroup with higher myeloma expression level had better prognosis. However, for gliomas of the central nervous system, the results were reversed^32^. The results^33,34^ of two pan-cancer studies suggest that FCER1G is involved in the immune infiltration process of tumors and may be a potential immunotherapeutic target. Our experiment confirmed that it was highly expressed in the sinuses of normal tissue adjacent tumor, while the expression of tumors was reduced. Kupffer Cells (KC) were widely present in normal liver tissues and were a key mediator of the inflammatory response that occurs within liver tissue cells. Several studies had demonstrated that KC was lowly expressed in hepatocellular carcinoma tissues^35^, while FCER1G protein was expressed in monocytes/macrophages, which was consistent with our study. Presenilin 1 (PSEN1) was an intramembrane protease, the active subunit of the γ-secretase complex. Presenilin-1 (PSEN1) and presenilin-2 (PSEN2) are two genes associated with several diseases, and early studies have focused on their association with familial Alzheimer's disease (FAD), which often leads to early onset of the disease^36–38^. Many recent studies had proved that PNEN1 was involved in the initiation and development of tumors and was always accompanied by bad effects^39,40^. KEGG showed that PNEN1, as an upstream protein, positively regulated the process of β- catenin protein entering the nucleus, which promoted the occurrence of tumors^41,42^. The histology of β-catenin activated hepatocellular adenoma might have moderate cytological and structural abnormalities, which is difficult to differentiate from highly differentiated HCC. This adenoma also has a higher malignancy rate, too. Many studies had proved that the continuous activation of Wnt/β-catenin signaling pathway make malignant tumor cells have the characteristics of continuous self-renewal and growth, which will also reduce the efficacy of HCC immunotherapy^43,44^. In studies focused on treatment, Ma et al.^45^ showed that high expression of PSEN1 increased the radiation resistance and chemotherapy tolerance of hepatocellular carcinoma cells. SLAM family member 6(SLAM6) belongs to the Signaling lymphocyte Activating molecule (SLAM) family. Its receptors regulate innate and adaptive immune responses and trigger cytolytic activity in some natural killer cells (NK)^46,47^. Our experiments found that SLAM6 protein expression levels were decreased in HCC. Study has shown that SLAMF6 promotes the development of liver cancer by promoting macrophage M2 polarization^48^. Other studies have shown that CD8^+^ T cell responses during chronic viral infections are sustained by interleukin-21 (IL-21) from CD4^+^ T cells, which are composed of three transcriptionally and epigenetically distinct populations: Cxcr5 + Tfh cells, Slamf6 + Memory-like (Tml) subsets, Cxcr6 + Th1 cells^49^. The positive correlation between Slamf6 and CD8^+^ T cell may explain the poor prognosis when the expression is decreased. In short, the four genes in the prognostic model are involved in the immune activity of the human body, further studying its mechanisms helps to provide new ideas for treatment.

Compared with other prognostic models, such as Fourteen-gene, Twelve-gene, Ten-gene, and Six-gene^50–53^. Our prediction model achieves more accurate predictions and higher C-index scores with fewer genes. Inevitably, individual studies had some limitations. The validity of this signature needs to be verified by more HCC samples. The mechanism of how each gene affects the immune efficacy needs more study. In addition, in vivo and in vitro assays will be added in future studies to further investigate the expression of these four genes at the protein level and prognostic relevance, including their roles in HCC progression.

## Conclusions

In summary, our study constructed an immunotherapy-related risk model for predicting prognosis and individualized immunotherapy for HCC patients, which was effective in classifying those patients.

### Supplementary Information

Supplementary Information.

## Data Availability

The datasets used and/or analyzed during the current study (TCGA-LIHC, ICGC-LIRI-JP, and GSE140901cohorts) are available from the corresponding author upon reasonable request.

## Abbreviations

HCC: Hepatocellular carcinoma
ICB: Immune checkpoint blockade
IHC: Immunohistochemistry
WGCNA: Weighted correlation network analysis
LASSO: Least absolute shrinkage and selection operator
GSEA: Gene set enrichment analysis
ICIs: Immune checkpoint inhibitors
ENG: Endoglin
FCER1G: Function of the high-affinity IgE receptor
IgE: Immunoglobulin E
KC: Kupffer cells
PSEN1: Presenilin 1
FAD: Familial Alzheimer’s disease
SLAM: Signaling lymphocyte activating molecule

## References

[1] Alvarez CS, Petrick JL, Parisi D, McMahon BJ, Graubard BI, McGlynn KA (2022). Racial/ethnic disparities in hepatocellular carcinoma incidence and mortality rates in the United States, 1992–2018. Hepatology.

[2] Xu LL, Zou C, Zhang SS, Chu TSM, Zhang Y, Chen WW (2022). Reshaping the systemic tumor immune environment (STIE) and tumor immune microenvironment (TIME) to enhance immunotherapy efficacy in solid tumors. J. Hematol. Oncol..

[3] Steven A, Fisher SA, Robinson BW (2016). Immunotherapy for lung cancer. Respirology.

[4] Morrison AH, Byrne KT, Vonderheide RH (2018). Immunotherapy and prevention of pancreatic cancer. Trends Cancer.

[5] Schakelaar, M. Y., Monnikhof, M., Crnko, S., Pijnappel, E., Meeldijk, J., Ten Broeke, T. *et al.* Cellular immunotherapy for medulloblastoma. *Neuro Oncol.* (2022). 10.1093/neuonc/noac236 PMC10076947 36219688

[6] Ouyang T, Kan X, Zheng C (2022). Immune checkpoint inhibitors for advanced hepatocellular carcinoma: Monotherapies and combined therapies. Front. Oncol..

[7] Wen, W., Zhang, Y., Zhang, H. & Chen, Y. Clinical outcomes of PD-1/PD-L1 inhibitors in patients with advanced hepatocellular carcinoma: A systematic review and meta-analysis. *J. Cancer Res. Clin. Oncol.* (2022). 10.1007/s00432-022-04057-3 PMC11797376 35771261

[8] Schmid AS, Neri D (2019). Advances in antibody engineering for rheumatic diseases. Nat. Rev. Rheumatol..

[9] Zhao QY (2017). On the indirect relationship between protein dynamics and enzyme activity. Prog. Biophys. Mol. Biol..

[10] Doroshow DB, Bhalla S, Beasley MB, Sholl LM, Kerr KM, Gnjatic S (2021). PD-L1 as a biomarker of response to immune-checkpoint inhibitors. Nat. Rev. Clin. Oncol..

[11] Hsu CL, Ou DL, Bai LY, Chen CW, Lin L, Huang SF (2021). Exploring markers of exhausted CD8 T cells to predict response to immune checkpoint inhibitor therapy for hepatocellular carcinoma. Liver Cancer.

[12] Barretina J, Caponigro G, Stransky N, Venkatesan K, Margolin AA, Kim S (2012). The Cancer Cell Line Encyclopedia enables predictive modelling of anticancer drug sensitivity. Nature.

[13] Zhou Y, Zhou B, Pache L, Chang M, Khodabakhshi AH, Tanaseichuk O (2019). Metascape provides a biologist-oriented resource for the analysis of systems-level datasets. Nat. Commun..

[14] Li T, Fan J, Wang B, Traugh N, Chen Q, Liu JS (2017). TIMER: A web server for comprehensive analysis of tumor-infiltrating immune cells. Cancer Res..

[15] Gentles AJ, Newman AM, Liu CL, Bratman SV, Feng W, Kim D (2015). The prognostic landscape of genes and infiltrating immune cells across human cancers. Nat. Med..

[16] Aran D, Hu ZC, Butte AJ (2017). xCell: Digitally portraying the tissue cellular heterogeneity landscape. Genome Biol..

[17] Tang Z, Li C, Kang B, Gao G, Li C, Zhang Z (2017). GEPIA: A web server for cancer and normal gene expression profiling and interactive analyses. Nucleic Acids Res..

[18] Sberna AL, Bouillet B, Rouland A, Brindisi MC, Nguyen A, Mouillot T (2018). European Association for the Study of the Liver (EASL), European Association for the Study of Diabetes (EASD) and European Association for the Study of Obesity (EASO) clinical practice recommendations for the management of non-alcoholic fatty liver disease: Evaluation of their application in people with Type 2 diabetes. Diabet. Med..

[19] Kudo M (2020). Recent advances in systemic therapy for hepatocellular carcinoma in an aging society: 2020 update. Liver Cancer.

[20] Sangro B, Gomez-Martin C, de la Mata M, Inarrairaegui M, Garralda E, Barrera P (2013). A clinical trial of CTLA-4 blockade with tremelimumab in patients with hepatocellular carcinoma and chronic hepatitis C. J. Hepatol..

[21] El-Khoueiry AB, Sangro B, Yau T, Crocenzi TS, Kudo M, Hsu C (2017). Nivolumab in patients with advanced hepatocellular carcinoma (CheckMate 040): An open-label, non-comparative, phase 1/2 dose escalation and expansion trial. Lancet..

[22] Zhu AX, Finn RS, Edeline J, Cattan S, Ogasawara S, Palmer D (2018). Pembrolizumab in patients with advanced hepatocellular carcinoma previously treated with sorafenib (KEYNOTE-224): A non-randomised, open-label phase 2 trial. Lancet Oncol..

[23] Ren ZG, Xu JM, Bai YX, Xu AB, Cang SD, Du CY (2021). Sintilimab plus a bevacizumab biosimilar (IBI305) versus sorafenib in unresectable hepatocellular carcinoma (ORIENT-32): A randomised, open-label, phase 2–3 study. Lancet Oncol..

[24] Yau T, Park JW, Finn RS, Cheng AL, Mathurin P, Edeline J (2019). CheckMate 459: A randomized, multi-center phase III study of nivolumab (NIVO) vs sorafenib (SOR) as first-line (1L) treatment in patients (pts) with advanced hepatocellular carcinoma (aHCC). Ann. Oncol..

[25] Philip M, Schietinger A (2022). CD8^+^ T cell differentiation and dysfunction in cancer. Nat. Rev. Immunol..

[26] Lee NY, Blobe GC (2007). The interaction of endoglin with beta-arrestin2 regulates transforming growth factor-beta-mediated ERK activation and migration in endothelial cells. J. Biol. Chem..

[27] Kasprzak A, Adamek A (2018). Role of endoglin (CD105) in the progression of hepatocellular carcinoma and anti-angiogenic therapy. Int. J. Mol. Sci..

[28] Jeng KS, Sheen IS, Lin SS, Leu CM, Chang CF (2021). The role of endoglin in hepatocellular carcinoma. Int. J. Mol. Sci..

[29] Chen XP, Chen YG, Lan JY, Shen ZJ (2014). MicroRNA-370 suppresses proliferation and promotes endometrioid ovarian cancer chemosensitivity to cDDP by negatively regulating ENG. Cancer Lett..

[30] Dong K, Chen W, Pan X, Wang H, Sun Y, Qian C (2022). FCER1G positively relates to macrophage infiltration in clear cell renal cell carcinoma and contributes to unfavorable prognosis by regulating tumor immunity. BMC Cancer.

[31] Hasan MZ, Walter L (2021). Rhesus macaque activating killer immunoglobulin-like receptors associate with fc receptor gamma (FCER1G) and not with DAP12 adaptor proteins resulting in stabilized expression and enabling signal transduction. Front. Immunol..

[32] Xu H, Zhu Q, Tang L, Jiang J, Yuan H, Zhang A (2021). Prognostic and predictive value of FCER1G in glioma outcomes and response to immunotherapy. Cancer Cell Int..

[33] Zhang X, Cai J, Song F, Yang Z (2022). Prognostic and immunological role of FCER1G in pan-cancer. Pathol. Res. Pract..

[34] Yang R, Chen Z, Liang L, Ao S, Zhang J, Chang Z (2023). Fc Fragment of IgE Receptor Ig (FCER1G) acts as a key gene involved in cancer immune infiltration and tumour microenvironment. Immunology.

[35] Matsuda M, Seki E (2020). Hepatic stellate cell-macrophage crosstalk in liver fibrosis and carcinogenesis. Semin. Liver Dis..

[36] Lanoiselee HM, Nicolas G, Wallon D, Rovelet-Lecrux A, Lacour M, Rousseau S (2017). APP, PSEN1, and PSEN2 mutations in early-onset Alzheimer disease: A genetic screening study of familial and sporadic cases. PLoS Med..

[37] Arber C, Lovejoy C, Harris L, Willumsen N, Alatza A, Casey JM (2021). Familial Alzheimer’s disease mutations in PSEN1 lead to premature human stem cell neurogenesis. Cell Rep..

[38] Kim YE, Cho H, Kim HJ, Na DL, Seo SW, Ki CS (2020). PSEN1 variants in Korean patients with clinically suspicious early-onset familial Alzheimer’s disease. Sci. Rep..

[39] Pan X, Zhao T, Mu S, Li S (2021). miR-193a directly targets PSEN1 and inhibits gastric cancer cell growth, the activation of PI3K/Akt signaling pathway, and the epithelial-to-mesenchymal transition. J. Oncol..

[40] Wei W, Zhang Y (2022). PSEN1 is associated with colon cancer development via potential influences on PD-L1 nuclear translocation and tumor-immune interactions. Front. Immunol..

[41] Killick R, Pollard CC, Asuni AA, Mudher AK, Richardson JC, Rupniak HT (2001). Presenilin 1 independently regulates beta-catenin stability and transcriptional activity. J. Biol. Chem..

[42] Chen Q, Schubert D (2002). Presenilin-interacting proteins. Expert Rev. Mol. Med..

[43] Du W, Menjivar RE, Donahue KL, Kadiyala P, Velez-Delgado A, Brown KL (2023). WNT signaling in the tumor microenvironment promotes immunosuppression in murine pancreatic cancer. J. Exp. Med..

[44] Yu F, Yu C, Li F, Zuo Y, Wang Y, Yao L (2021). Wnt/beta-catenin signaling in cancers and targeted therapies. Signal Transduct. Target Ther..

[45] Ma H, Yuan L, Li W, Xu K, Yang L (2018). The LncRNA H19/miR-193a-3p axis modifies the radio-resistance and chemotherapeutic tolerance of hepatocellular carcinoma cells by targeting PSEN1. J. Cell Biochem..

[46] Bottino C, Falco M, Parolini S, Marcenaro E, Augugliaro R, Sivori S (2001). NTB-A [correction of GNTB-A], a novel SH2D1A-associated surface molecule contributing to the inability of natural killer cells to kill Epstein-Barr virus-infected B cells in X-linked lymphoproliferative disease. J. Exp. Med..

[47] Eissmann P, Watzl C (2006). Molecular analysis of NTB-A signaling: A role for EAT-2 in NTB-A-mediated activation of human NK cells. J. Immunol..

[48] Meng Q, Duan X, Yang Q, Xue D, Liu Z, Li Y (2022). SLAMF6/Ly108 promotes the development of hepatocellular carcinoma via facilitating macrophage M2 polarization. Oncol. Lett..

[49] Zander R, Kasmani MY, Chen Y, Topchyan P, Shen J, Zheng S (2022). Tfh-cell-derived interleukin 21 sustains effector CD8^+^ T cell responses during chronic viral infection. Immunity.

[50] Zhang BH, Yang J, Jiang L, Lyu T, Kong LX, Tan YF (2020). Development and validation of a 14-gene signature for prognosis prediction in hepatocellular carcinoma. Genomics.

[51] Ouyang G, Yi B, Pan G, Chen X (2020). A robust twelve-gene signature for prognosis prediction of hepatocellular carcinoma. Cancer Cell Int..

[52] Liang JY, Wang DS, Lin HC, Chen XX, Yang H, Zheng Y (2020). A novel ferroptosis-related gene signature for overall survival prediction in patients with hepatocellular carcinoma. Int. J. Biol. Sci..

[53] Liu GM, Zeng HD, Zhang CY, Xu JW (2019). Identification of a six-gene signature predicting overall survival for hepatocellular carcinoma. Cancer Cell Int..

