# Supplementary material for: A novel tumor immunotherapy-related signature for risk stratification, prognosis prediction, and immune status in hepatocellular carcinoma
Source: Sci Rep. 2023 Oct 31;13:18709. doi: 10.1038/s41598-023-46252-3 (PMC10618198; doi:10.1038/s41598-023-46252-3)
Supplement: Supplementary file 1 — Supplementary Information. [file 41598_2023_46252_MOESM1_ESM.docx]

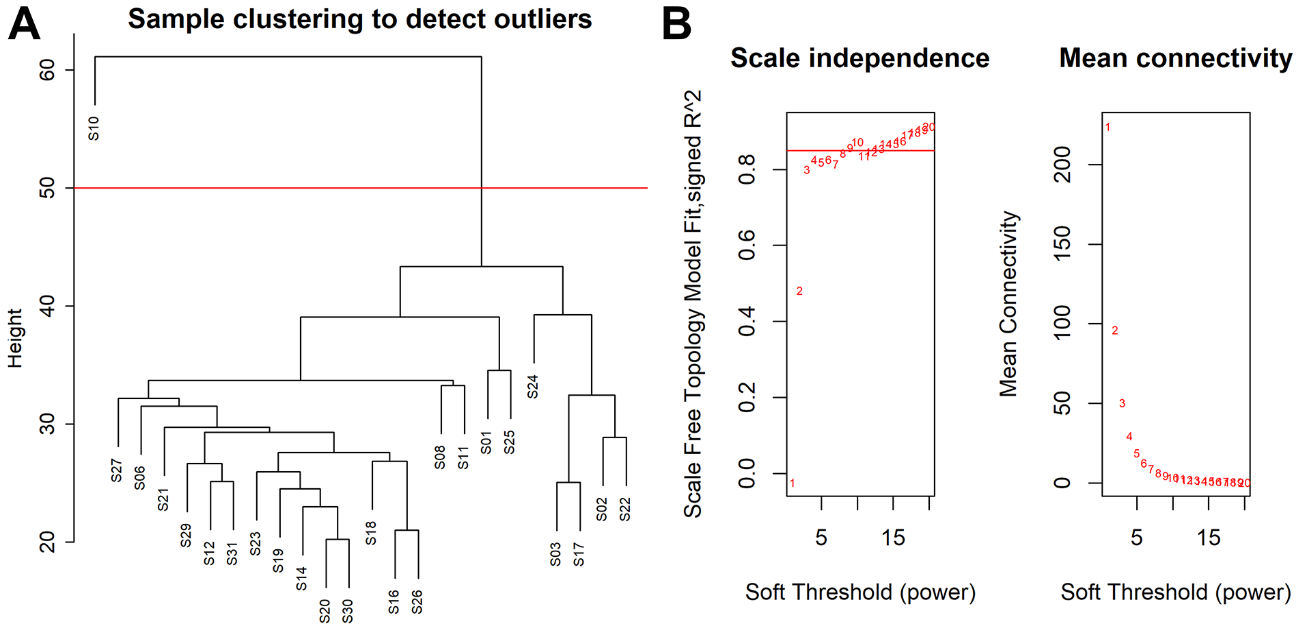


Figure S1. Cluster analysis of HCC samples and Power value screening of genes in HCC co-expression modules. (A) Clustering dendrogram of the clinical traits and data from 24 HCC samples, the outliers are removed. (B) Analysis of network topology for various soft-thresholding powers (weighting coefficient, *β*). when the power value was 9, the independence degree was ≥0.85.


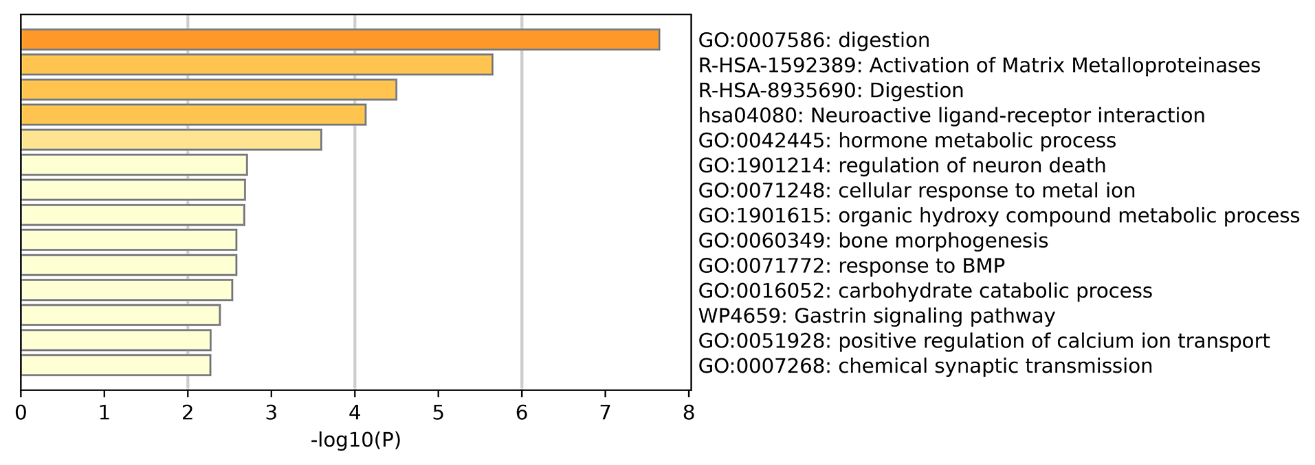


Figure S2. Enrichment analysis of immunotherapy-related genes with different risk scores.


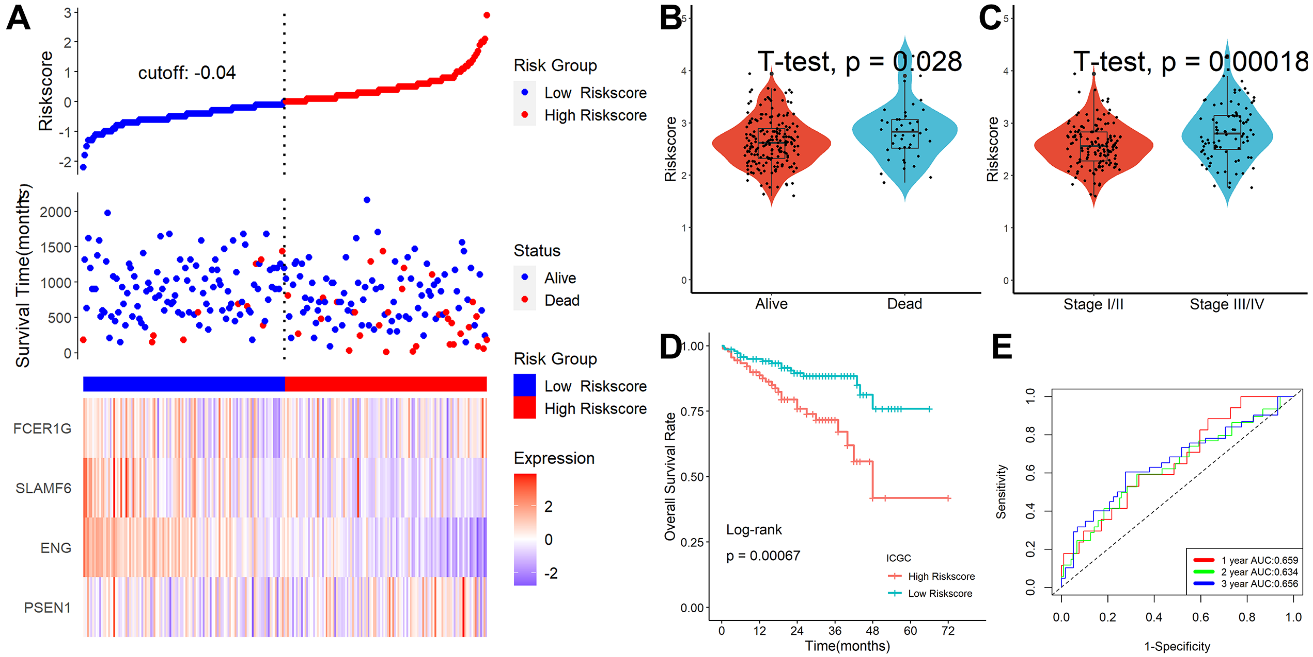


Figure S3. Validation of the signature in ICGC. (A)The distribution of risk scores, OS status, and expression profiles in ICGC cohort. (B) Patients with higher risk scores had significantly decreased survival outcomes in the ICGC cohorts. (C) Different risk scores have different TNM stages. (D) Kaplan-Meier survival plot. (E) ROC analysis predicts prognostic value of 1-, 2-, and 3-year OS rates.


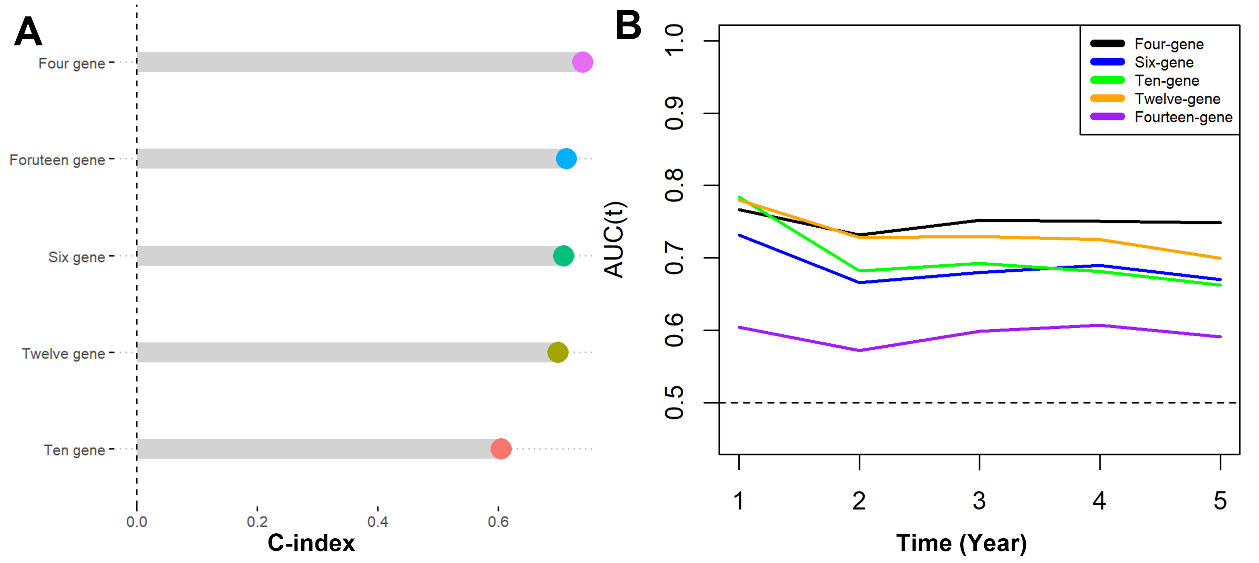


Figure S4. Compared with previous research results.

Table S1 Clinical characteristics of HCC patients involved in the study

|  | TCGA cohort  (N=365) | ICGC cohort  (N=227) | Validation cohort |
| --- | --- | --- | --- |
| Gender Male | 119 | 61 | 23 |
| Female | 246 | 166 | 7 |
| Age ≤60 years | 173 | 49 | 22 |
| >60 years | 192 | 178 | 8 |
| Grade G1/2 | 230 |  | 14 |
| G3/4 | 130 |  | 16 |
| unknown | 5 |  |  |
| TNM Stage I/II | 254 | 140 | 11 |
| III/IV | 87 | 87 | 19 |
| unknown | 24 | 0 |  |
| Vascular Invasion Yes | 106 |  | 11 |
| No | 205 |  | 19 |
| unknown | 5 |  |  |
| Recurrence With tumor | 122 |  |  |
| Tumor free | 161 |  |  |
| unknown | 82 |  |  |
| Cirrhosis With | 68 |  |  |
| Without | 141 |  |  |
| unknown | 156 |  |  |
| HBV or HCV Infection |  |  |  |
| Yes | 149 |  |  |
| No | 203 |  |  |
| unknown | 13 |  |  |
| Child-Pugh A | 216 |  |  |
| B | 21 |  |  |
| C | 1 |  |  |
| unknown | 127 |  |  |

Table S2 RiskScore of our Nomogram

| var | time | days | cutoff | tp | fp | ppv | npv | sensitivities | specificities |
| --- | --- | --- | --- | --- | --- | --- | --- | --- | --- |
| riskScore | 1 | 365 | 1.6760461 | 0.65426721 | 0.145 | 0.43300722 | 0.9359447 | 0.65426721 | 0.855 |
| riskScore | 2 | 730 | 1.51518924 | 0.63681664 | 0.19090909 | 0.49983981 | 0.88145995 | 0.63681664 | 0.80909091 |
| riskScore | 3 | 1095 | 1.43285258 | 0.71603701 | 0.15068493 | 0.64723521 | 0.8856663 | 0.71603701 | 0.84931507 |
| riskScore | 4 | 1460 | 0.80226246 | 0.87189674 | 0.28846154 | 0.59151477 | 0.9205959 | 0.87189674 | 0.71153846 |
| riskScore | 5 | 1825 | 1.29214911 | 0.69205804 | 0.14705882 | 0.71821165 | 0.83644458 | 0.69205804 | 0.85294118 |
